# ZCMM: A Novel Method Using Z-Curve Theory- Based and Position Weight Matrix for Predicting Nucleosome Positioning

**DOI:** 10.3390/genes10100765

**Published:** 2019-09-28

**Authors:** Ying Cui, Zelong Xu, Jianzhong Li

**Affiliations:** 1 College of Computer Science and Technology, Heilongjiang University, Harbin 150080, China; cuiying204@163.com; 2 College of Bioinformatics Science and Technology, Harbin Medical University, Harbin 150081, China; xykcdzd@163.com; 3 College of Computer Science and Technology, Harbin Institute of Technology, Harbin 150001, China

**Keywords:** nucleosome positioning, Z-curve, position weight matrix, support vector machine

## Abstract

Nucleosomes are the basic units of eukaryotes. The accurate positioning of nucleosomes plays a significant role in understanding many biological processes such as transcriptional regulation mechanisms and DNA replication and repair. Here, we describe the development of a novel method, termed ZCMM, based on Z-curve theory and position weight matrix (PWM). The ZCMM was trained and tested using the nucleosomal and linker sequences determined by support vector machine (SVM) in Saccharomyces cerevisiae (*S. cerevisiae*), and experimental results showed that the sensitivity (*Sn*), specificity (*Sp*), accuracy (*Acc*), and Matthews correlation coefficient (*MCC*) values for ZCMM were 91.40%, 96.56%, 96.75%, and 0.88, respectively, and the average area under the receiver operating characteristic curve (AUC) value was 0.972. A ZCMM predictor was developed to predict nucleosome positioning in *Homo sapiens* (*H. sapiens*)*, Caenorhabditis elegans* (*C. elegans*)*,* and *Drosophila melanogaster* (*D. melanogaster*) genomes, and the accuracy (*Acc*) values were 77.72%, 85.34%, and 93.62%, respectively. The maximum AUC values of the four species were 0.982, 0.861, 0.912 and 0.911, respectively. Another independent dataset for *S. cerevisiae* was used to predict nucleosome positioning. Compared with the results of Wu’s method, it was found that the *Sn*, *Sp*, *Acc,* and *MCC* of ZCMM results for *S.* *cerevisiae* were all higher, reaching 96.72%, 96.54%, 94.10%, and 0.88. Compared with the Guo’s method ‘iNuc-PseKNC’, the results of ZCMM for *D. melanogaster* were better. Meanwhile, the ZCMM was compared with some experimental data in vitro and in vivo for *S. cerevisiae*, and the results showed that the nucleosomes predicted by ZCMM were highly consistent with those confirmed by these experiments. Therefore, it was further confirmed that the ZCMM method has good accuracy and reliability in predicting nucleosome positioning.

## 1. Introduction

The basic unit of eukaryotic chromatin is the nucleosome. Each nucleosome contains a 147-bp core DNA that is tightly wrapped in 1.67 left-handed super-helical turns around a histone octamer. Composed of DNA and a protein core, nucleosomes are about 10 nm in diameter and are the fundamental repeating unit of the chromatin structure of eukaryotic DNA [1,2,3,4]. Nucleosome tissues play a crucial role in controlling the DNA accessibility of many DNA-binding proteins to regulatory elements on chromosomes [5]. Nucleosome positioning broadly indicates where nucleosomes are located with respect to genomic DNA sequences [6]. By modulating the accessibility of genomic regions to regulatory proteins, it was observed that the packaging of DNA around the histone octamer plays important roles in many biological processes [7,8]. Most studies on nucleosome function have indicated that nucleosomes are involved in chromatin formation [9], antagonistic transcription factors [10,11], and regulating gene expression [12,13].

Chromatin at active gene promoters is characterized by a distinct nucleosome [14]. Nucleosomal sequences play a crucial role in controlling the DNA accessibility of many DNA-binding proteins to regulatory elements on chromosomes [5]. Knowing the accurate locations of nucleosomes within a genome is key to understanding gene regulation; however, the correct location and positioning of the nucleosome within the genome has been a long-standing issue in chromatin biology [15]. Precise nucleosome localization affects gene expression regulation, DNA replication, DNA repair, and DNA recombination [16,17,18,19]. It is important to investigate the mechanism involved in controlling nucleosome localization. The most common view is that nucleosome localization is determined by a combination of DNA sequences, ATP-dependent remodeling enzymes, transcription factors, and prolonged RNA polymerases [9,20]. DNA sequence features have been considered as important contributors to nucleosome assembly. The usefulness of nucleosome studies depends on the accuracy and precision of the map of nucleosomes in vivo. A number of different protocols have been developed to map nucleosome locations genome-wide, but currently it is common to digest chromatin fibers using micrococcal nuclease (MNase) followed by high-throughput sequencing (MNase-seq). The mechanism and method for yeast nucleosome localization have been proposed, indicating that nucleosomes can be guided by DNA in the coding region of the eukaryotic genome [21,22]. Some scholars proposed using sequencing of DNase I hypersensitive sites (DNase-seq) [23] to study nucleosome localization. It has also been proposed that circulating nucleosomes could be used as cancer markers, as nucleosomes that occupy certain parameters could serve as the starting point for replication [24,25,26].

Studying nucleosome positioning mainly concentrates on prediction algorithms, such as the algorithm with information entropy [7], the bases of deflection angle method to represent the nucleosome DNA sequence characteristics [13,26,27], methods to train the support vector machine (SVM) [22,28,29,30,31,32,33], the Bayesian algorithm [34], and neural network classifiers [35,36]. While nucleosome positioning is cell-type-dependent, the DNA sequence still plays an important role in directing preferential histone octamer assembly. Teif summarized the available experimental datasets, computational tools to analyze nucleosome positioning data and computational tools to predict nucleosome positioning from DNA sequences [37]. There are several methods for predicting nucleosome positioning based on sequence features that have been put forth in recent years, such as iNuc-PhysChem [38], iNuc-PseKNC [36], and nuMap [39]. Nucleosome positioning identified by computational methods is a valuable complement to experimental verification.

In this study, we propose a novel method, termed ZCMM, which is based on the Z-curve theory and position weight matrix (PWM). The Euclidean distance between the samples and the model was calculated as the feature, which was put into the support vector machine (SVM) to train and test the model for predicting nucleosomes. Z-curve theory was put forward by Zhang [40]; it is a novel method for mapping DNA or RNA sequences into a folding curve in three-dimensional space. The Z-curve has been proposed based on the symmetry of regular tetrahedrons. There exists a unique Z-curve for a given DNA sequence; therefore, a DNA sequence can be uniquely determined by a given Z-curve. The properties of Z-curves have been studied in great detail. It is well known that PWM is a model used to represent transcriptional factor binding sites based on the base features. In our paper, we constructed a Z-curve theory model (Z-curve model) based on a sequence and a PWM model for a dataset of nucleosomal sequences. Then, the ZCMM model was established by putting forward the Z-curve model multiplied by the PWM, which extracted the feature of nucleosomal and linker sequences, and provided the features for the SVM so as to predict nucleosome positioning across different species, such as Saccharomyces cerevisiae (*S. cerevisiae*)*, Homo sapiens* (*H. sapiens*)*, Caenorhabditis elegans* (*C. elegans*)*,* and *Drosophila melanogaster* (*D. melanogaster*) genomes. The experimental results show that the ZCMM method can achieve good predictive efficiency and has strong stability.

## 2. Materials and Methods 

### 2.1. Benchmark Datasets for Nucleosomal and Linker Sequences 

In this study, we first targeted *S. cerevisiae*. The benchmark dataset S_1_ for *S. cerevisiae* was selected from Chou’s work [38]. The reference genome sequence of *S. cerevisiae* was obtained from the Saccharomyces Genome Database (http://www.yeastgenome.org/). The nucleosome positions of *S. cerevisiae* were derived from the published data obtained by Lee al. [41], where each of the 1,206,683 DNA fragments in the dataset constructed by these authors was assigned a nucleosome formation score using a lasso model, with a high or low score to reflect its high or low propensity in forming nucleosome, respectively. The low score can also be interpreted as the propensity to inhibit the formation of nucleosomes. To prepare a high-quality benchmark dataset, 5000 fragments of 150 bp with the highest scores were selected as the nucleosome-forming sequence samples to construct the positive dataset, and 5000 fragments of 150 bp with the lowest scores were selected as the nucleosome-inhibiting (or linker) sequence samples to construct the negative dataset.

Another dataset S_2_ was obtained from Wei Chen’s work [42], including nucleosomal sequences and linker sequences, and CD-HIT software [38] was used with the cutoff threshold set at 80% to remove redundancies. The two datasets can be mathematically expressed as:

(1) $$\left\{ \begin{array}{l} S_{1}=S_{1}^{+}+S_{1}^{-}\\ S_{2}=S_{2}^{+}+S_{2}^{-} \end{array}\right.\;S.\;\mathrm{cerevisiae}$$

In Formula (1), the dataset $S_{1}$ contains 10,000 samples, of which 5000 are nucleosomal sequences belonging to the positive subset $S_{1}^{+}$ and 5000 are linker sequences belonging to the negative subset $S_{1}^{-}$. $S_{2}$ contains 3620 samples of 150 bp, of which 1880 are nucleosomal sequences belonging to the positive subset $S_{2}^{+}$ and 1740 are linker sequences belonging to the negative subset $S_{2}^{-}$. 

Three other different species were considered in this paper, i.e., *H. sapiens*, *C. elegans*, and *D. melanogaster*. The benchmark datasets for these species were taken from Guo’s work [36]. The experimental data for nucleosome positions in the first species [43] were downloaded from http://dir.nhlbi.nih.gov/papers/lmi/epigenomes/hgtcellnucleosomes.aspx; those for the second species from http://hgdownload.cse.ucsc.edu; and those for the third species from [44,45] and http://atlas.bx.psu.edu/. The entire genome sequences for the three species were downloaded from the UCSC (University of California Santa Cruz) Genome Browser: http://hgdownload.cse.ucsc.edu/, where the hg18 version, WS170/ce4 version and BDGP Release5 version were used for (1) *H. sapiens*, (2) *C. elegans*, and (3) *D. melanogaster* genomes, respectively [46]. CD-HIT software [47] was applied to remove redundant samples from benchmark datasets with a cutoff threshold value of 80%. These datasets were obtained as formulated by:

(2) $$\left\{ \begin{array}{l} S_{H}=S_{H}^{+}+S_{H}^{-},\;H.\;\mathrm{sapiens}\\ S_{C}=S_{C}^{+}+S_{C}^{-},\;C.\;\mathrm{elegans}\\ S_{D}=S_{D}^{+}+S_{D}^{-}.\;D.\;\mathrm{melanogaster} \end{array}\right.$$

In Formula (2), the length of the all sequences for the three species is 147 bp. The dataset S_H_ contains 4573 samples, of which 2273 are nucleosome-forming sequences belonging to the positive subset $S_{H}^{+}$ and 2300 are nucleosome-inhibiting sequences belonging to the negative subset $S_{H}^{-}$. Meanwhile, S_C_ contains 5175 samples, of which 2567 are nucleosome-forming sequences belonging to the positive subset $S_{C}^{+}$ and 2608 are nucleosome-inhibiting sequences belonging to the negative subset $S_{C}^{-}$. S_D_ contains 5750 samples, of which 2900 are nucleosome-forming sequences belonging to the positive subset $S_{D}^{+}$, and 2850 are nucleosome-inhibiting sequences belonging to the negative subset $S_{D}^{-}$. 

### 2.2. Construction of the ZCMM Model for Nucleosomal and Linker Sequences

The Z-curve theory was put forward by Zhang [40]; it is a novel method for mapping DNA or RNA sequences into a folding curve in three-dimensional space. The Z-curve was proposed based on the symmetry of regular tetrahedrons. There exists a unique Z-curve for each given DNA sequence; therefore, a DNA sequence can be determined by a given Z-curve. The properties of Z-curves have been studied in great detail. The Z-curve theory is a relatively complete, systematic theory for investigating DNA sequences [48], internationally recognized and widely applied for DNA sequence analysis and gene identification. The Z-curve has three components: x, y, and z, each with a specific biological significance [40]. x denotes the distribution of purine (A + G) to pyrimidine (C + T) along the sequences. When the number of purine bases is more than that of pyrimidine bases, x >0; otherwise x <0; when these two are equal, x = 0. Similarly, y denotes the distribution of amino (A + C) to ketone (G + T) along the sequences. z denotes the distribution of weak hydrogen bonds (A + T) to strong hydrogen bonds (G + C) along the sequence. The three components of Z-curve contain the information of distribution of the bases in the DNA sequence.

In this study, we propose a novel model, termed ZCMM, for nucleosome positioning, which integrates the Z-curve theory [49] and position weight matrix to perform nucleosome localization. The ZCMM model is constructed by the following four steps.

In step 1, each nucleosomal or linker sequence is transformed to a matrix of 3*N by normalization Formula (3), which is converted into N ordered points in space. $a_{n}$, $c_{n}$, $g_{n}$ and $t_{n}$ represent the cumulative frequency of bases A, G, C, and T in the subsequence from the 1^st^ to the n^th^ position in a single sequence. The $x_{n}$, $y_{n}$ and $z_{n}$ rows of the matrix represent the three coordinate components, and their range is between −1.0 and 1.0. $x_{n}$ represents the distribution of purine (A + G) to pyrimidine (C + T) of one sequence along the DNA sequence; $y_{n}$ represents the distribution of amino (A + C) to ketone (G + T) of one sequence along the DNA sequence; and $z_{n}$ represents the distribution of weak hydrogen bonds (A + T) to strong hydrogen bonds (G + C) of one sequence along the DNA sequence [40,49].

A single DNA sequence is transformed by Formula (3) as follows:

(3) $$\begin{array}{l} \left\{ \begin{array}{l} x_{n}=(a_{n}+g_{n})-(c_{n}+t_{n})\\ y_{n}=(a_{n}+c_{n})-(g_{n}+t_{n})\\ z_{n}=(a_{n}+t_{n})-(g_{n}+c_{n}) \end{array}\right.\\ n=1,2,\ldots ,N. \end{array}$$

In step 2, for a set of nucleosomal sequences or linker sequences from the first to the nth position, the mean value of their corresponding positions is calculated to obtain the Z-curve model (ZCM) by Formula (4). $a_{n}^{m}$, $g_{n}^{m}$, $c_{n}^{m}$ and $t_{n}^{m}$ represent the average frequencies of accumulation of the four bases A, G, C, and T from the 1^st^ to the n^th^ position for m nucleosomal or linker sequences. $x_{n}$,$y_{n}$, and $z_{n}$ are the same as in step 1. The ZCM represents the distribution of the average base accumulation frequencies of m sequences in the horizontal direction of DNA sequence. The ZCM is an accumulation of characteristic information, which can comprehensively represent the characteristics of nucleosomal or linker sequences, while the preference of m sequences in a single position in the vertical direction is rarely considered.

A set of nucleosomal or linker sequences for the ZCM is transformed by Formula (4) as follows:

(4) $$\begin{array}{l} \left\{ \begin{array}{l} x_{n}=(a_{n}^{m}+g_{n}^{m})-(c_{n}^{m}+t_{n}^{m})\\ y_{n}=(a_{n}^{m}+c_{n}^{m})-(g_{n}^{m}+t_{n}^{m})\\ z_{n}=(a_{n}^{m}+t_{n}^{m})-(g_{n}^{m}+c_{n}^{m}) \end{array}\right.\\ n=1,2,\ldots ,N. \end{array}$$

In step 3, the position weight matrix (PWM) is constructed for a set of the nucleosomal or linker sequences by Formula (5). Here, $q_{in}$ represents the frequency of the bases *i* (A, T, G, C) in the *n*th (1,2, …, 147) position of the nucleosome DNA. $b_{i}$ is a background frequency that represents the frequency of bases *i* occurring genome-wide. M is a matrix of 4*N and each element is denoted as $M_{in}$. The PWM model represents the base preference of m sequences at a single position in the vertical direction.

(5) $$\begin{array}{l} M_{in}=\log\left(\frac{q_{in}}{b_{i}}\right)\;i\in \left\{A,T,G,C\right\}\;n=1,2,\ldots ,N.\\ M=\left[\begin{array}{l} M_{A1}\;M_{A2}\;\ldots \;M_{AN}\\ M_{G1}\;M_{G2}\;\ldots \;M_{GN}\\ M_{C1}\;M_{C2}\;\ldots \;M_{CN}\\ M_{T1}\;M_{T2}\;\ldots \;M_{TN} \end{array}\right] \end{array}$$

In step 4, a new combination model—ZCMM—is constructed by Formula (6), and the ZCMM model is established by putting forward the ZCM model from Formula (4) multiplying M by Formula (5). Thus, the element value $M_{in}$ of M is multiplied by the corresponding element values in the Z-curve model for the three coordinates, as shown in Formula (6). ZCMM is a coordinate matrix of 3*N, and contains more feature information for identifying nucleosome positioning in the genome. The PWM element value put into Z-curve model is employed as a new weight in ZCMM. In this way, the ZCMM model not only reflects the distribution of the base accumulation frequency in the horizontal direction, but also reflects the preference of the base distribution in the vertical direction. Therefore, the ZCMM model can better represent the characteristics of nucleosomal sequences or linker sequences than the Z-curve model or the PWM, in theory. We use experimental data to confirm this in the results.

Consider that the Z-curve model represents the distribution of the average base accumulation frequency of m sequences in the horizontal direction of DNA sequences. We can understand the ZCMM model in this way, wherein *x_n_* represents the difference between the weighted cumulative content of (A + G) and the weighted cumulative content of (C + T); *y_n_* represents the difference between the weighted cumulative content of (A + C) and the weighted cumulative content of (G + T); *z_n_* represents the difference between the weighted cumulative content of (A + T) and the weighted cumulative content of (G + C). 

A set of nucleosomal or linker sequences for the ZCMM model is transformed by Formula (6) as follows:

(6) $$\begin{array}{l} \left\{ \begin{array}{l} X_{n}=(M_{n}^{A}\times a_{n}^{m}+M_{n}^{G}\times g_{n}^{m})-(M_{n}^{C}\times c_{n}^{m}+M_{n}^{T}\times t_{n}^{m})\\ Y_{n}=(M_{n}^{A}\times a_{n}^{m}+M_{n}^{C}\times c_{n}^{m})-(M_{n}^{G}\times g_{n}^{m}+M_{n}^{T}\times t_{n}^{m})\\ Z_{n}=(M_{n}^{A}\times a_{n}^{m}+M_{n}^{T}\times t_{n}^{m})-(M_{n}^{G}\times g_{n}^{m}+M_{n}^{C}\times c_{n}^{m}) \end{array}\right.\\ n=1,2,\ldots ,N \end{array}$$

### 2.3. ZCMM Method for Identifying Nucleosome Positioning by SVM 

The Euclidean distance is calculated to represent the similarity between a single nucleosomal or linker sequence and the ZCMM model at each position by Formula (7). $Ed_{n}$ is the Euclidean distance at the *n*th position, and $x_{n}$, $y_{n}$, and $z_{n}$ are calculated by Formula (3). $X_{n}$, $Y_{n}$ and $Z_{n}$ are calculated by Formula (6). $D^{m}$ is the Euclidean distance vector between the *m*^th^ nucleosomal or linker sequence and the ZCMM model. *N* and *M* are respectively the length and number of nucleosomal or linker sequences. Finally, two Euclidean distance matrices are calculated as a positive subset and a negative subset for nucleosomal or linker sequences.

(7) $$\begin{array}{l} Ed_{n}^{m}=\sqrt{{\left(x_{n}-X_{n}\right)}^{2}+{\left(y_{n}-Y_{n}\right)}^{2}+{\left(z_{n}-Z_{n}\right)}^{2}}\\ n=1,\;2,\;\ldots ,\;N.\\ D^{m}=\left[Ed_{1}^{m},Ed_{2}^{m},\;\ldots ,\;Ed_{N}^{m}\right]\\ m=1,\;2,\;\ldots ,\;M. \end{array}$$

Support vector machines (SVMs) are currently a hot topic in the machine learning community, creating a similar enthusiasm at the moment as artificial neural networks used to do before. SVMs represent a powerful technique for general (nonlinear) classification, regression and outlier detection with an intuitive model representation. SVM is a powerful and popular method for pattern recognition that has been widely used in the realm of bioinformatics. In this article, the “e1071” package was used for the implementation of SVM, which can be downloaded from https://cran.rstudio.com/web/packages/e1071/index.html. The package “e1071” offers an interface to the award-winning C++ implementation by Chang and Lin. For further implementation details on libsvm, see a practical guide to SVM classification (https://www.csie.ntu.edu.tw/~cjlin/libsvm/). The predictor obtained via the above procedures is called the ZCMM method, which is a sequence-based predictor for nucleosome positioning, integrating the Z-curve theory and position weight matrix. Further, ZCMM can distinguish between nucleosomal and linker sequences by training and testing the positive distance matrix and negative distance matrix. The prediction method ZCMM is based on two parameters, namely gamma and cost in the R package “e1071”. Therefore, we searched for the optimal values of the two parameters, ensuring better results for predicting the nucleosome sequences. 

### 2.4. A Set of Metrics for Quantitative Analysis

In the statistical prediction model, a fundamental task is the partition of provided data into training and testing subsets. In the literature, the cross-validation test is extensively applied for evaluating the quality and effectiveness of the developed model. Here, 10-fold cross-validation is applied, in which the data are divided into 10-fold, where nine parts used for the training process and the remaining is utilized for the testing process. The same process is repeated 10 times. Finally, the outcome is yielded on the basis of the average. The metrics for measuring the prediction performance are mathematically expressed by Formula (8):

(8) $$\left\{ \begin{array}{ll} S_{n}=1-\frac{N_{-}^{+}}{N^{+}} & 0\leq S_{n}\leq 1\\ Sp=1-\frac{N_{+}^{-}}{N^{-}} & 0\leq S_{p}\leq 1\\ Acc=1-\frac{N_{-}^{+}+N_{+}^{-}}{N^{+}+N^{-}} & 0\leq Acc\leq 1\\ MCC=\frac{1-\left(\frac{N_{-}^{+}+N_{+}^{-}}{N^{+}+N^{-}}\right)}{\sqrt{\left(1+\frac{N_{+}^{-}-N_{-}^{+}}{N^{+}}\right)\left(1+\frac{N_{-}^{+}-N_{+}^{-}}{N^{-}}\right)}} & -1\leq MCC\leq 1 \end{array}\right.$$

where $N^{+}$ is the total number of positive samples or nucleosomal sequences investigated, while $N_{-}^{+}$ is the number of nucleosomal sequences incorrectly predicted to be linker sequences. $N^{-}$ is the total number of negative samples or linker sequences investigated, while $N_{+}^{-}$ is the number of linker sequences incorrectly predicted to be nucleosomal sequences [50]. Formula (8) is widely utilized to compute the prediction of classifiers. Here, there are four parameters [39], including the sensitivity (*Sn*), specificity (*Sp*), total accuracy (*Acc*), and Matthew correlation coefficient (*MCC*). These four metrics are generally used in statistical prediction to quantitatively measure the performance of a predictor from four different angles. In some statistical analyses, *Sn* is also called the “true positive rate” and (*1 − Sp*) the “false positive rate”. The area under the receiver operating characteristic curve (auROC or AUC) was also calculated to evaluate the classification effectiveness of the ZCMM method.

## 3. Results

### 3.1. ZCMM Model Construction and Statistical Analysis

**Figure 1:**
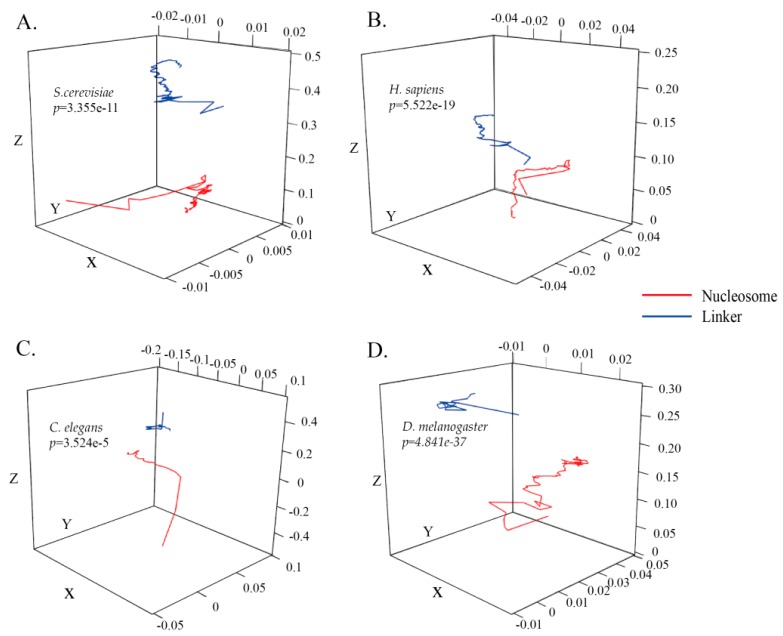
Three-dimensional space imaging of nucleosomal and linker sequences using our model for four species. (**A**–**D**) represent Saccharomyces cerevisiae (*S. cerevisiae*)*, Homo sapiens* (*H. sapiens*)*,* *Caenorhabditis elegans* (*C. elegans*) and *Drosophila melanogaster* (*D. melanogaster*), respectively. The red curve represents the nucleosomal sequence model and the blue curve represents the linker sequence model.

Nucleosomal sequences as the positive subset and linker sequences as the negative subset in *S. cerevisiae* were used to construct the ZCMM models. Image projection of the two ZCMM models along the *X*, *Y,* and *Z* axes was drawn by R software, and the difference between the two imaging projections was examined by the Wilcoxon rank-sum test in Figure 1A and supplementary Figure S1A–C. From the perspective of the coordinate distribution range, the *x* and *y* coordinate distribution range is small (<0.01) in Figure 1A (supplementary Figure S1A,B), while the *z* coordinate distribution range is large, ranging from 0.0 to 0.5 in Figure 1 (supplementary Figure S1C). According to the formula established by the model, it indicates that the total content of (A + G) and (C + T) as well as the total content of (A + C) and (T + G) in the nucleosomal sequences or linker sequences have a smaller difference, while the content of (A + T) and (C + G) in the nucleosomal or linker sequences has a larger difference. There are also differences between the *X*, *Y*, and *Z* curves in Figure 1A (supplementary Figure S1A–C). Meanwhile, from the perspective of the curve variation trend, the nucleosomal sequences and linker sequences have different variation trends along the DNA sequence in the coordinate system. In particular, it was found that the *p*-value for the global 3D curve images at 150 corresponding positions between the nucleosomal dataset and linker dataset for *S. cerevisiae* was less than 0.01 (*p*-value as shown in Figure 1A). The 3D images of *H. sapiens*, *C. elegans,* and *D. melanogaster* are respectively shown in Figure 1B–D, respectively. The *p*-values for these three species, determined by the Wilcoxon rank-sum test, were less than 0.01 (*p*-values were shown in Figure 1B–D). The results of these three species were similar to those found for *S. cerevisiae*. The ZCMM model could reflect the characteristics of nucleosomal sequences and linker sequences, and could distinguish between the two categories significantly in four species. This is beneficial for the further improvement of the efficiency of the classifier.

Furthermore, we randomly selected a nucleosomal sequence and a linker sequence to be converted into a Z-curve to analyze the differences between the two. The *p*-values of the global 3D curves including the three dimensions of *x*, *y,* and *z* were less than 0.01 (all *p*-values were shown in supplementary Figure S2). Therefore, it was significantly different between nucleosomal and linker sequence models, and the constructed model ZCMM reflected the characteristic differences between nucleosomes and linkages in statistics.

### 3.2. ZCMM Model Compared with Z-Curve Model and PWM Model

**Figure 2:**
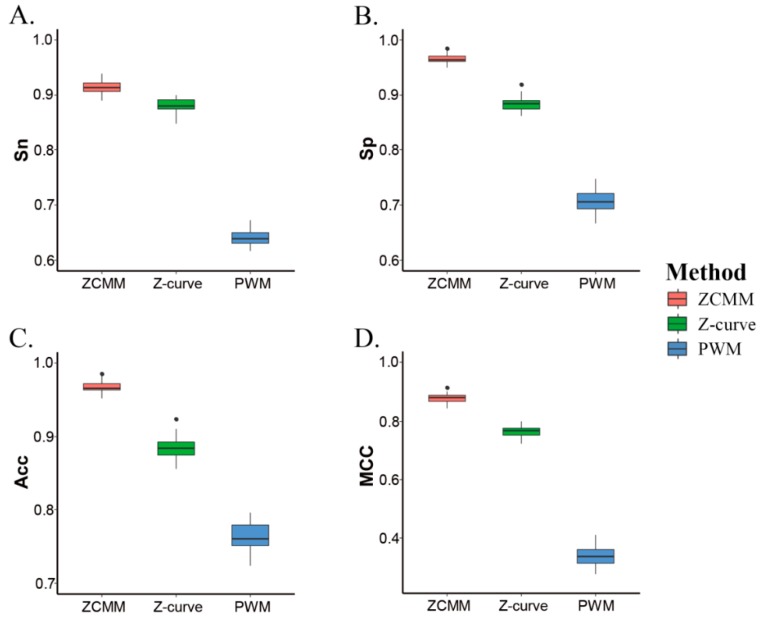
Comparison of four performances of three models for prediction results by 10-fold cross validation of support vector machine (SVM). (**A**–**D**) represent *Sn*, *Sp*, *Acc*, and *MCC*, respectively.

In order to better analyze model performances, we compared the ZCMM model with the Z-curve model and the position weight matrix (PWM) model for predicting nucleosomal sequences by the SVM method (10-fold cross-validation) in *S. cerevisiae* ($S_{1}$). Experimental results are shown in Table 1. Values for the four parameters (*Sn*, *Sp*, *Acc,* and *MCC*) of the ZCMM model were respectively 91.40%, 96.56%, 96.75%, and 0.88, which were higher than the results of the Z-curve model and PWM model. All results of the 10-fold cross-validation are shown in Figure 2. The results show that ZCMM, obtained by integrating the Z-curve and PWM models, is superior to both the Z-curve and PWM model alone in terms of the *Sn*, *Sp*, *ACC,* and *MCC*. More complete nucleosomal sequence information or linker sequence information can be obtained by considering the two factors in ZCMM. This provided a guarantee for the predicting nucleosomal sequences by ZCMM.

### 3.3. ZCMM Prediction Results in Different Species

We applied ZCMM to predict nucleosomal sequences for multiple species, i.e., *S. cerevisiae*, *H. sapiens, C. elegans,* and *D. melanogaster*, which used the datasets $S_{1}$, *S*_H_, *S*_C_, and *S*_D_ with 10-fold cross validation. Experimental results are shown in Table 2. 

In the *S. cerevisiae* genome, the *Sn*, *Sp*, *Acc*, *MCC* and average AUC were 91.40%, 96.56%, 96.75%, 0.88, and 0.972. In *H. sapiens*, the *Sn* was 74.87%, the average AUC was 0.851, and the *Acc* and *MCC* values were less than 0.8. This may be related to both the strong dynamics of the human whole-genome nucleosome and the small dataset of human nucleosome sequences in our study. In *C. elegans*, the *Sp*, *Acc,* and average AUC were respectively 84.10%, 85.34%, and 0.872, while the *Sn* and *MCC* were less than 80% and 0.7. In *D. melanogaster*, the *Sp*, *Acc* and average AUC were respectively 92.26%, 93.62%, and 0.877; the *Sn* and *MCC* were about 80% and 0.7. The two groups of *C. elegans* and *D. melanogaster* were low, possibly because in the model training process, in order to reduce the computational time, we trained over less time and obtained relatively better parameters rather than the optimal parameters. The overall performance of ZCMM was good. 

**Figure 3:**
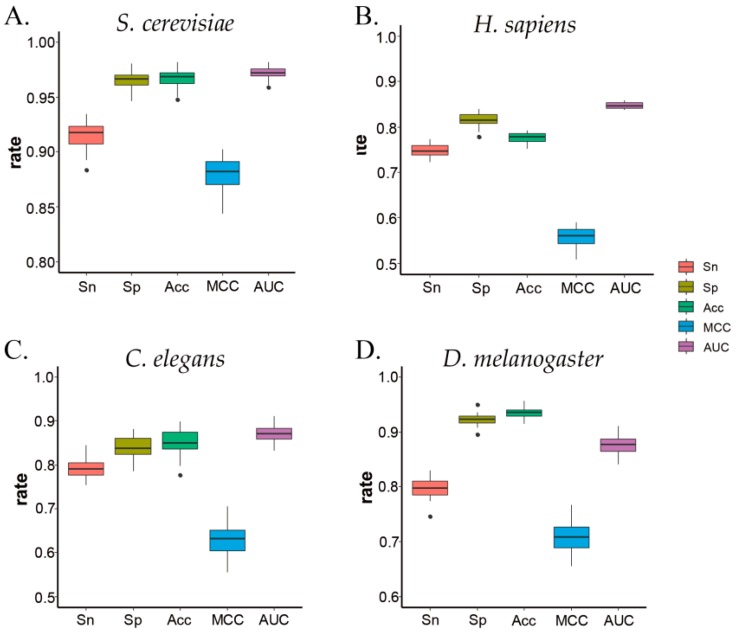
The metrics of prediction by 10-fold cross-validation. (**A**–**D**) represent the results for *S. cerevisiae, H. sapiens, C. elegans,* and *D. melanogaster*, respectively.

Meanwhile, to verify the performance of each prediction result, a 2D box figure was drawn for each 10-fold cross-validation in the four species. The four species and five indicators are shown in Figure 3. As a whole, the distribution of all experimental results was relatively concentrated near the average with a small fluctuation range, which means that our ZCMM method has good stability.

**Figure 4:**
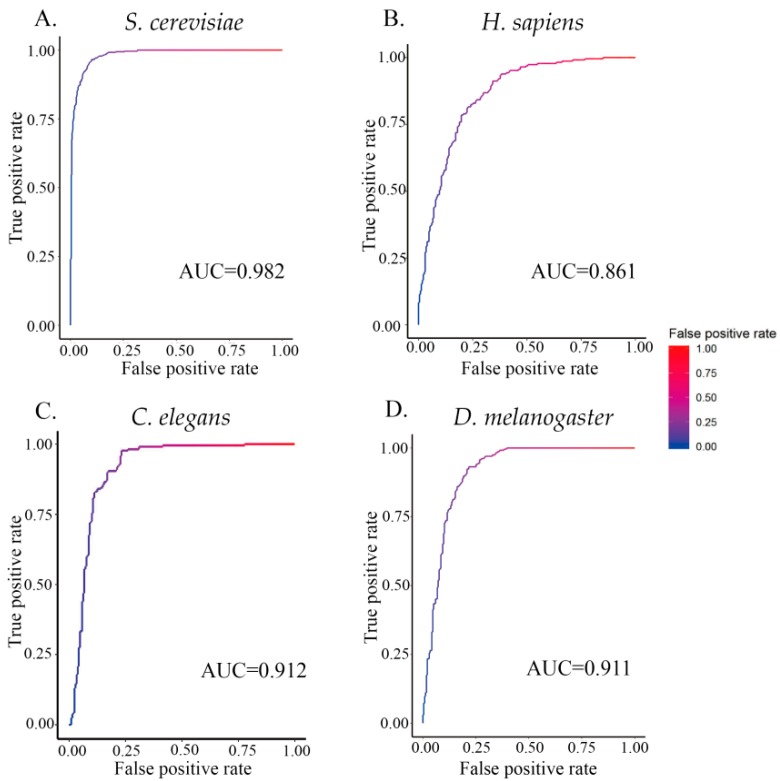
AUC obtained from 10-fold cross-validation. (**A**–**D**) represent the maximum AUC or areas under the ROC curve (0.982, 0.861, 0.912, and 0.911) for *S. cerevisiae, H. sapiens, C. elegans,* and *D. melanogaster*, respectively.

Furthermore, to provide a graphical illustration showing the performance of the classifier ZCMM, a 2D plot, called the receiver operating characteristic (ROC) curve, is given in Figure 3. In this figure, the vertical coordinate *Y* is for the true positive rate or *Sn*, while the horizontal coordinate *X* is the false positive rate or (1 – *Sp*). The AUC (area under the curve) is defined as the area under the ROC curve (auROC). This parameter is often used to indicate the performance quality of a classifier; the AUC value of 0.5 of is equivalent to random prediction, while the value of 1.0 represents a perfect prediction. The AUC values for the four species by ZCMM method are given in Figure 4, and the maximum AUC values of four species were 0.982, 0.861, 0.912, and 0.911 for *S. cerevisiae, H. sapiens,* *C. elegans,* and *D. melanogaster*, respectively. These results further show that our method has good classification efficiency. 

### 3.4. Performance Compared with Other Methods

There are many methods for nucleosome prediction based on sequence features. Wu’s method, which is also based on the Z-curve theory, and iNuc-PseKNC, which is a sequence-based predictor of nucleosome positioning, were selected to be compared with our ZCMM method for three species, as shown in Table 3. 

According to Wu’s algorithm ideas [51], we used R language programming to complete the experiment. The results of iNuc-PseKNC were obtained from Guo’s paper [36]. From the four performance values of *Sn*, *Sp*, *Acc*, and *MCC*, the ZCMM method in three species was shown to be generally superior to Wu’s method. For the *H. sapiens* genome, compared to the iNuc-PseKNC method, the *Sn* value obtained by ZCMM was higher, and the *Sp*, *Acc,* and *MCC* of ZCMM were a little lower. For the *C. elegans* genome, the four performance values of ZCMM were a little lower than those found by the iNuc-PseKNC method. For the *D. melanogaster* genome, the *Sp*, *Acc,* and *MCC* of ZCMM were higher than those obtained by iNuc-PseKNC. As the four indexes of the ZCMM method were almost consistently better than those obtained by Wu’s method, ZCMM was compared with the iNuc-PseKNC method in terms of the ROC curve area in Table 3. The AUC value of *H. sapiens* using ZCMM was 0.861, which was a little lower than that obtained by iNuc-PseKNC. The AUC value of *C. elegans* using ZCMM was 0.912, close to value of 0.925 obtained by iNuc-PseKNC. The AUC value of *D. melanogaster*, according to ZCMM, was 0.911—this was higher than the value found by iNuc-PseKNC (0.874). This result indicates that the ZCMM prediction method can be extended to more species and still maintain good accuracy after considering the position weight matrix in the Z-curve model.

### 3.5. Performance Validation Using Independent Data for S. cerevisiae 

In order to validate the performance of the ZCMM model, we used dataset S_1_ (5000 nucleosomal and 5000 linker sequences) and S_2_ of *S. cerevisiae* (1740 nucleosomal and 1880 linker sequences). The results are shown in Table 4. Our ZCMM model obtained efficient outcomes—its four performance values were much higher than those obtained by Wu’s method, proving that the success rates of the ZCMM model are more efficient. Thus, the ZCMM model shows the characteristics of nucleosomal and linker sequences very well, and it was found that our ZCMM method not only achieved good classification verification, but also had strong stability. 

### 3.6. Occupancy Profile of Predicting Nucleosomal Sequences

**Figure 5:**
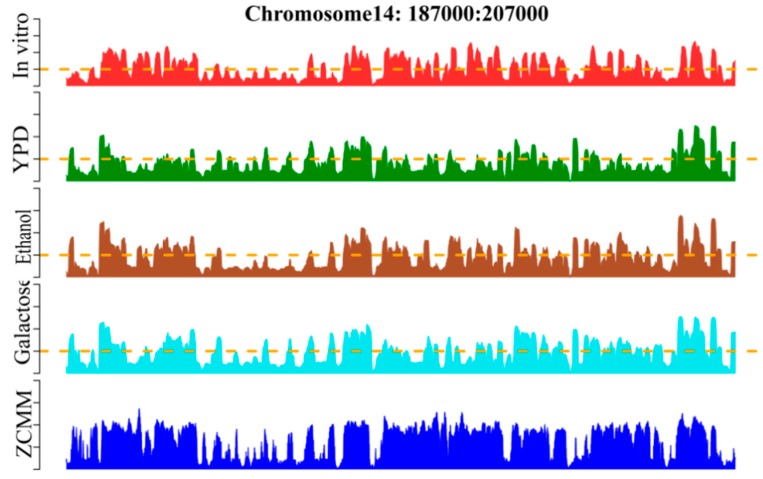
Illustration to show the nucleosome occupancy profile predicted by the ZCMM model on *S. cerevisiae* chromosome 14 (187000:207000). To facilitate the comparison, the corresponding profiles by experiments are also shown. In a top–down order, they are the genomic position on the chromosome, the experimental map in vitro, and the experimental maps in vivo for YPD, Ethanol, and Galactose, respectively. Depicted in the lowest panel is the nucleosome occupancy profile determined by the proposed ZCMM.

Whole-genome nucleosome prediction was performed by the sliding window method across *S. cerevisiae* using the ZCMM algorithm. We compared the prediction results with those of other experiments proving the nucleosome in vitro and in vivo in chromosome 14 (see Figure 5). The genomic position on the chromosome, the experimental map in vitro [52,53], and the experimental maps in vivo [2,7] for YPD, Ethanol, and Galactose are given in the figure. We found that the profile of nucleosome occupancy predicted by ZCMM was notably similar to the experimental maps of nucleosome positioning [54]. Similar peaks and valleys appeared in the graphs and their positions were very consistent, demonstrating once again that our ZCMM is indeed a very good approach for predicting nucleosome positioning.

Furthermore, we used Pearson correlation to calculate the similarity values of ZCMM prediction results and the other four experimental results, and the correlation coefficients were 0.67, 0.36, 0.57 and 0.50, respectively. This indicates that our prediction model can accurately predict the occupancy rate of nucleosomes, and may help to find the potential locations of nucleosomes in a given genome. ZCMM could be used to predict nucleosome occupancy for whole genome.

## 4. Discussion

In this study, the ZCMM predictor is proposed for the prediction of nucleosome positioning in genomes. In this predictor, the ZCMM model integrates the Z-curve theory and PWM to extract the features of nucleosomal and linker sequences, and the extracted feature spaces provided by ZCMM are presented by the SVM to identify nucleosome positioning for different species, namely *S. cerevisiae, H. sapiens, C. elegans* and *D. melanogaster* genomes. The experimental results show that the ZCMM method can achieve good predictive efficiency and has strong stability. The nucleosome occupancy profiles predicted by the ZCMM method are agreement with many other experimental results in the *S. cerevisiae* genome. Furthermore, we consider that the ZCMM method, which integrates the Z-curve and position weight matrix, can fully demonstrate the features of the sequences not only in the horizontal direction, but also in the vertical direction of the sequences. It can also better show features of nucleosomal and linker sequences. Therefore, the ZCMM was found to be useful and have good accuracy, and could be as a better novel method for the recognition and prediction of nucleosome localization. Nucleosome localization is a complex process; it still needs to be further studied, though the research on nucleosome positioning has seen some progress. Our work not only provides a new method for nucleosome positioning, but also reveals that the primary DNA sequence is an important factor of nucleosome formation. Overall, this study contributes to the comprehension of the pattern of nucleosome positioning and nucleosome occupancy in genomes.

Still, the features extracted from the nucleosome data are not comprehensive enough, so the final prediction effect is affected. In future, we will further study the localization of nucleosomes. On the one hand, we will collect much more standard datasets to conduct nucleosome classification prediction, so as to verify the effectiveness of our feature model, and ultimately improve the prediction accuracy of the model. On the other hand, we plan to consider training the model based on deep learning, and furthermore extract more updated features and improve the prediction performances. Finally, a prediction system of nucleosome location will be developed to provide a service for other researchers.

## Figures and Tables

**Table 1:** 10-fold cross-validation of different methods.

Species	*Sn%*	*Sp%*	*Acc%*	*MCC*
ZCMM	91.40	96.56	96.75	0.88
Z-curve	88.15	88.40	88.42	0.77
PWM	63.97	70.67	76.32	0.34

*Sn*: sensitivity, *Sp*: specificity, *Acc*: total accuracy, *MCC*: Matthew correlation coefficient (*MCC*). ZCMM: our model Z-curve theory-based and position weight matrix, Z-curve: model based on Z-curve theory, PWM: model based on position weight matrix.

**Table 2:** Prediction results of 10-fold cross-validation for four species by ZCMM

Species	*Sn*%	*Sp*%	*Acc*%	*MCC*	AUC
*S. cerevisiae* (*S*_1_)	91.40	96.56	96.75	0.88	0.972
*H. sapiens*	74.87	81.51	77.72	0.56	0.851
*C. elegans*	78.80	84.10	85.34	0.62	0.872
*D. melanogaster*	79.64	92.26	93.62	0.70	0.877

AUC: Area under the receiver operating characteristic curve.

**Table 3:** Performance compared with other methods.

Species	Method	*Sn*%	*Sp*%	*Acc*%	*MCC*	AUC
*H. sapiens*	ZCMM	74.87	81.51	77.72	0.56	0.861
	Wu’s method	65.82	64.12	64.74	0.30	
	iNuc-PseKNC	86.27	87.86	84.70	0.73	0.925
*C. elegans*	ZCMM	78.80	84.10	85.34	0.62	0.912
	Wu’s method	89.90	71.64	76.05	0.62	
	iNuc-PseKNC	86.90	90.30	83.55	0.74	0.935
*D. melanogaster*	ZCMM	79.64	92.26	93.62	0.70	0.911
	Wu’s method	79.17	41.07	57.34	0.22	
	iNuc-PseKNC	79.97	78.31	81.65	0.60	0.874

AUC values of the three species by ZCMM are the maximum of 10-fold cross -validation.

**Table 4:** ZCMM compared with Wu’s method in two datasets of *S. cerevisiae.*

Dataset	Method	Sn%	Sp%	Acc%	*MCC*
S_1_	ZCMM	91.40	96.56	96.75	0.88
	Wu’s method	85.73	86.62	86.52	0.72
S_2_	ZCMM	96.72	96.54	94.10	0.88
	Wu’s method	86.20	84.89	85.57	0.71

## Supplementary Materials

The following are available online at https://www.mdpi.com/2073-4425/10/10/765/s1, Figure S1: Statistical test of our models of nucleosomal and linker sequences by visualization for *S. cerevisiae* (Wilcoxon rank-sum test), Figure S2: Statistical test between a random nucleosomal and a random linker sequence by visualization for *S. cerevisiae* (Wilcoxon rank-sum test).
