# Supplementary material for: ZCMM: A Novel Method Using Z-Curve Theory- Based and Position Weight Matrix for Predicting Nucleosome Positioning
Source: Genes (Basel). 2019 Sep 28;10(10):765. doi: 10.3390/genes10100765 (PMC6827144; doi:10.3390/genes10100765)
Supplement: Supplementary file 1 [file genes-10-00765-s001.pdf]

genes

Supplementary Materials: ZCMM: A Novel Method Using Z-Curve Theory- Based and Position Weight Matrix for Predicting Nucleosome Positioning

Ying Cui, Zelong Xu and Jianzhong Li

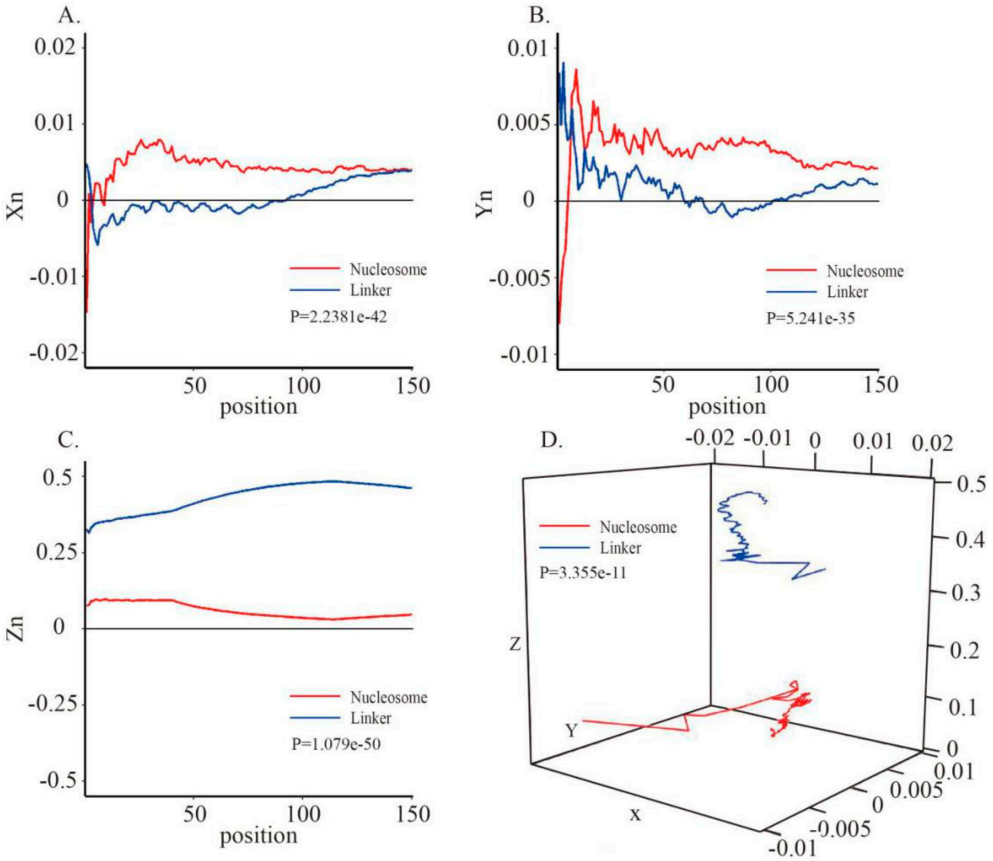

Figure S1. Statistical test of our models of nucleosomal and linker sequences by visualization for *S. cerevisiae* (Wilcoxon rank-sum test).

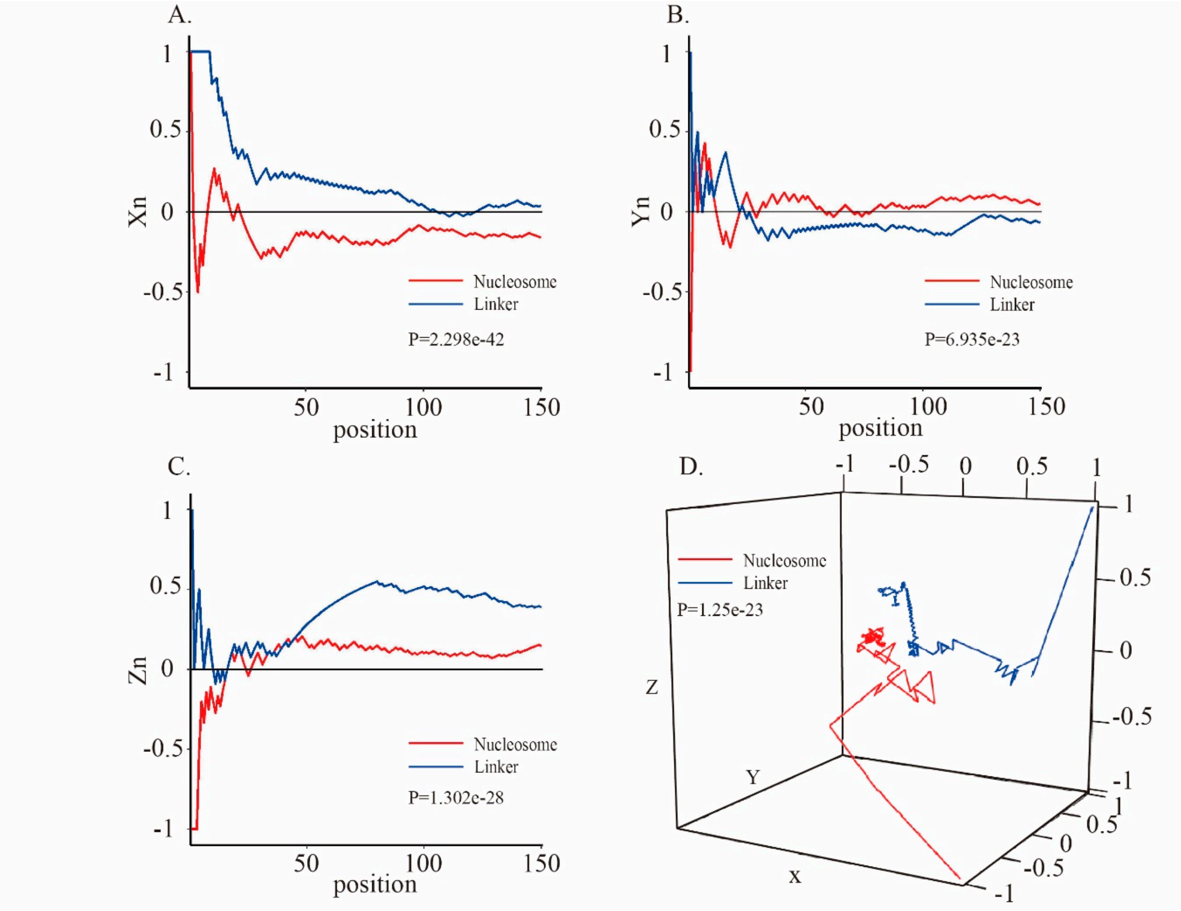

Figure S2. Statistical test between a random nucleosomal and a random linker sequence by visualization for *S. cerevisiae* (Wilcoxon rank-sum test).

© 2019 by the author. Licensee MDPI, Basel, Switzerland. This article is an open access article distributed under the terms and conditions of the Creative Commons Attribution (CC BY) license (http://creativecommons.org/licenses/by/4.0/).
